# Systematic CKD Care Approaches: A Potential Solution for the Costly ESRD Program

**DOI:** 10.1016/j.xkme.2022.100581

**Published:** 2022-12-09

**Authors:** Tonya Saffer, Alefiyah Mesiwala

**Affiliations:** 1 Outset Medical; 2 Former Senior Medical Director, UPMC Health Plan and Former ESCO Lead, Centers for Medicare and Medicaid Innovation

## Abstract

Care of patients with advanced kidney disease includes dialysis, kidney transplant, vascular access, primary care, and other specialist care, which are often siloed among multiple physicians, dialysis clinics, vascular access centers, and health system or hospital-based transplant programs. Other than the patient themselves, no one provider has holistic patient visibility or responsibility. Given that hospitals often lose money on Medicare patients who require dialysis services, momentum from innovation in advanced kidney care management, new technology with the potential for reduced costs, expansion of Medicare Advantage, and Medicare incentives for home dialysis could be leveraged by health systems to ultimately reduce the nearly $50 billion annual Federal spending on patients with kidney failure in the United States. Health systems, which offer many primary and specialty care services, may be uniquely positioned to leverage the more favorable economics associated with these changes to move kidney care from siloed, provider-centric care to integrated, patient-centric care. With 60% of patients initiating dialysis through an unplanned hospitalization, a holistic health system approach that includes offerings of kidney care management and kidney replacement therapy could move financial incentives away from the interests of any single provider and toward better addressing the total needs and the goals of the patient.

## Introduction

For the first time since 1972, when legislation was passed to provide Medicare coverage to nearly all Americans with permanent kidney failure requiring kidney replacement therapy, kidney care is embarking on a renaissance. A federal focus on improving kidney care, coupled with unprecedented investment in start-up companies in kidney care management, diagnostics and home dialysis promises to shift kidney care in the United States from a dialysis-centric focus to more holistic kidney care. For example, in 2020, two US-based kidney care companies raised millions with successful initial public offerings, one in early-stage analytics and the other a home hemodialysis machine, the first approved in over 15 years and only the second available in the United States.^1^^,^^2^ Globally, a successful initial public offering was launched by a company manufacturing a clinical diagnostic test to improve kidney transplant care.^3^ In addition, venture capital firms have invested millions into several new kidney care management companies to slow the progression of chronic kidney disease (CKD) and reduce the costs to health insurers of the transitions to kidney failure, with traditional dialysis organizations also beginning to develop relationships and partnerships to achieve this goal.^4^

These innovations in technology and kidney care management, lifted by significant policy changes, have been taking hold since 2015. Recent activity by Congress to expand Medicare Advantage (MA) enrollment to all dialysis patients and new reimbursement incentives led by the Centers for Medicare & Medicaid Services (CMS) have created momentum toward value-based kidney care.

According to the US Renal Data Systems, the mean expenditure for dialysis patients is approximately $88,000 per year with roughly a third attributed to dialysis, with higher costs, approximating $114,000 in total expenditures, during the first year of dialysis. Despite these costs, dialysis patients in the United States experience high mortality and hospitalization rates, 170 and 165 events respectively per 1,000 patient-years, and although nearly 30% of kidney failure patients have a functioning transplant, only a small fraction, fewer than 3% annually, receive a kidney transplant.^5^ Payers, dialysis providers, and kidney care companies are currently working together to improve earlier identification and management of CKD, delay progression to kidney failure, and reduce hospitalizations. However, a greater opportunity through integrated care models remains. Improving transitions between stages of kidney disease, inpatient and outpatient settings, home and transplant modalities, and improving overall patient health while also reducing the total expenditures on this complex population requires more than coordination between siloed entities responsible for narrow aspects of each patient’s care. Although health systems through hospitals tend to acute dialysis needs, these systems are also often managing patients with earlier stage CKD through primary care clinics. Therefore, they may be uniquely positioned to leverage innovative kidney care technology and care management strategies to implement quality improvement solutions that may reduce costs and close gaps between kidney care and patients’ holistic health care needs.

## Embracing Innovation Can Empower Health Systems to Lower Dialysis Costs

**Figure 1:**
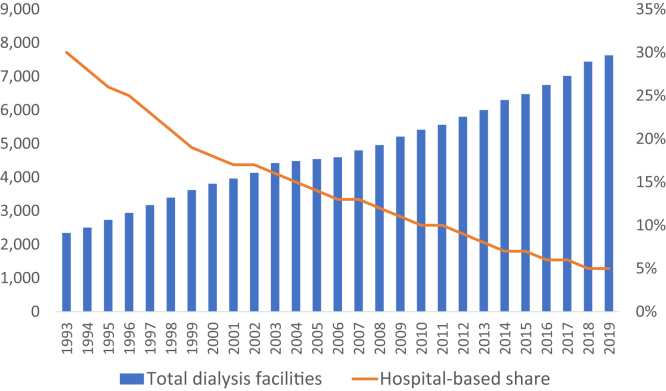
Dialysis facilities, 1993-2019. Abbreviation: Avg, average.

Kidney care needs health systems, which we define as inclusive of integrated health networks, accountable care organizations, and similar type health care organizations. Despite opportunity and existing infrastructure, many of these health systems have turned over both acute and long-term kidney care to outpatient dialysis chains, generating a direct funnel of acute hospitalized dialysis patients to these chain-owned outpatient dialysis clinics. Over the past 30 years, the number of hospital-based dialysis facilities declined from 30% of all dialysis locations to under 5% (Fig 1).

In addition, during this time, many hospitals began to outsource their acute dialysis programs to the large national dialysis providers optimized to deliver efficient and standardized treatment protocols.

However, recent kidney care innovations may enable health systems to establish acute and maintenance in-house kidney care services by providing analytics, specialized care management, and simplified dialysis equipment, reducing infrastructure and the number of skilled staff hours needed to treat patients.

Starting with insourcing acute dialysis, one recent example of a health system that made this shift was a 316-bed multispecialty acute care hospital in Salt Lake City, Utah. This hospital leveraged new technology and insourced all inpatient dialysis treatments, including staff, equipment, and dialysis device maintenance. Data regarding training time and treatment costs were recorded from April through November 2020 across the 577 treatments conducted and 15 providers. Comparing outsourced dialysis provider fees to the hospital in the prior year (annual cost of $650,000) to the new in-sourced model (annual cost of $200,000), an annual savings of $450,000 was projected for the first year alone because of the improved efficiency and productivity of providing dialysis internally.^6^

Given that many health systems treat kidney patients throughout their health care journey for routine primary and urgent care, are moving toward or have integrated electronic medical records, and have structured population health and interdisciplinary care programs, they may be well positioned to include long-term kidney care management, including preventive and tertiary care such as outpatient dialysis. By leveraging existing electronic medical record infrastructure and partnering with kidney care management companies or hiring experts, health systems can provide kidney care services to their existing patient population within their own system. Many examples of leveraging analytics, electronic medical records, and primary care providers have been shown to reduce unplanned or emergent dialysis initiation, increase home dialysis use, and achieve proactive dialysis access placement. Lastly, for health systems offering kidney transplantation services, managing CKD patients before kidney failure may allow them to increase transplants preemptively, increase living donor transplants, and potentially increase deceased donor transplants coming through their program.

For health systems considering starting their own kidney care service line, it is critical to evaluate both clinical and financial factors at 3 key points in the patient journey: dialysis initiation during an inpatient stay, outpatient dialysis care, and kidney transplant.

### Dialysis Initiation During Inpatient Stay

Currently, a patient who initiates maintenance dialysis while admitted to the hospital often experiences a lengthy inpatient stay before being discharged to a community-based dialysis clinic. An analysis performed by Health Management Associates of the 2016-2018 Medicare Limited Data Set showed the average hospital loses $8,500 per patient dialysis initiation, with more significant losses tied to lengths of stay beyond 10 days (Table 1). In this analysis, Medicare patients with *International Classification of Diseases, Tenth Revision* diagnosis of end-stage renal failure who received dialysis as an inpatient as the first event in 2017 but did not receive dialysis in 2016 were included, and patients who did not receive dialysis for at least 90 days after initiation were removed. The top diagnosis related groups in which these dialysis initiations occurred were then analyzed to determine revenue/loss by length of stay.

**Table 1:** Hospital Payment and Costs for Urgent Start Medicare Patients, 2017-2018

	% of Medicare Inpatient Data Files Starts	Medicare Inpatient Data Files Payment	Medicare Inpatient Data Files Cost	Medicare Inpatient Data Files Margin
2 days or less	7.1%	10,143	6,953	3,190
3 to 5 days	21.7%	14,875	11,313	3,561
6 to 8 days	24.2%	15,488	16,796	-1,308
9 to 10 days	11.7%	19,522	25,593	-6,071
11 to 12 days	8.5%	20,397	33,386	-12,989
13 to 15 days	10.1%	20,101	35,312	-15,212
16 to 20 days	7.6%	23,778	48,471	-24,693
21 days or more	9.1%	52,330	95,844	-43,514
**Total**		**$20,326**	**$28,840**	**-$8,515**

There is opportunity for a health system to minimize or eliminate these losses through the operation of its own outpatient dialysis program. With appropriate coordination between the acute and maintenance dialysis care teams, health systems may be able to discharge patients from inpatient status sooner, turning a 6-8 day stay at a loss into a 3-5 day stay with a positive operating margin.

The United States has an expensive and fragmented kidney care system with a patient experience defined by facility-based dialysis and stagnant clinical outcomes. Although survival was improving before coronavirus disease 2019, annual mortality rates have been largely flat over the past 5 years.^5^ Medicare spends 7% of its budget caring for the less than 2% of individuals with permanent kidney failure, and although Medicare spending on their dialysis care may be less than other countries, the private sector caring for the population enrolled in MA or commercial coverage spends approximately 3 times more than the public sector.^5^^,^^7^^,^^8^

**Figure 2:**
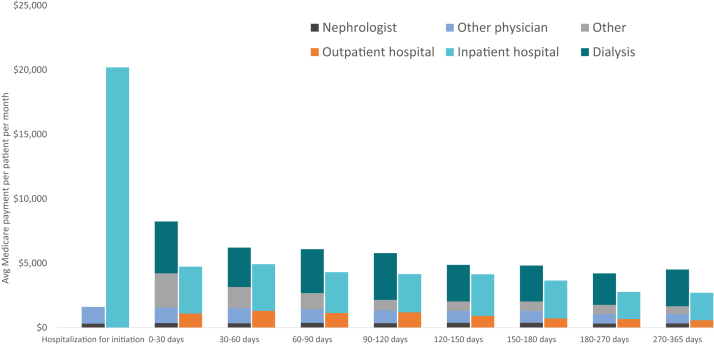
Average Medicare spending on patients initiating dialysis, 2017-2018. Source: Health Management Associates analysis of 2017-2019 5% Medicare claims data.

The Centers for Disease Control and Prevention report that CKD impacts about 15% of Americans, yet 40% of those with severe kidney disease are unaware that they have kidney disease. This lack of awareness is often associated with high CKD-related mortality or the surprising onset of permanent kidney failure. In 2017-2018, 57% of Medicare beneficiaries with incident kidney failure started dialysis urgently in the hospital, including 14% with no nephrologist visit before kidney failure and many more without enough nephrologist engagement to potentially change the trajectory of their disease. As shown in Fig 2, and is often a repeated anecdote by patients, new dialysis patients often find themselves navigating among the dialysis facility, hospitals, emergency rooms, access centers, nephrologists, and other clinical specialists. Medicare dialysis patients are hospitalized on average 1.6 times per year, spend 11 days in the hospital, and have a 35% readmission rate.^5^ For Medicare patients who began dialysis in 2017, per patient per year spending, in the first year, averaged $114,000 (including $42,000 inpatient, $9,800 outpatient hospital, and $36,000 outpatient dialysis). Despite this increased spending in the first year, annual mortality rates among prevalent dialysis patients have remained near 18% for the last 5 years.^5^ The number of patients dialyzing at home has increased slightly over the past decade, but at 13% of the dialysis population remains well behind many other developed countries.^5^

Dialysis patients require treatment at regular intervals to achieve adequate outcomes. Missed treatments double the risk of emergency department visits and quadruple the risk of hospitalization.^9^ Dialysis patients typically have 1-2 inpatient stays per year and an emergency department utilization rate that is 6-8 times greater than the general population.^9^ Hospitals are penalized by Medicare under the value-based purchasing program and hospital readmission reduction program and may lose money on the subsequent rehospitalizations.

Medicare fee-for-service spends approximately $36,000 per patient per year on dialysis treatment through a fixed bundled payment rate, but as mentioned, commercial payers often pay more, in part given limited negotiation power because of a highly consolidated industry.^7^ Assuming the average health system has 50 dialysis patients needing maintenance dialysis and a primary payer mix of 20% commercial at 2.5 times the Medicare rate, 50% Original Medicare and 30% MA at 1.14 times the Medicare rate, we estimate a health system’s dialysis treatment reimbursement could be $13.9 million for over 5 years (Table 2). This payer mix is reflective of the predicted incident dialysis population payer mix given that, as of 2021, dialysis patients were able to enroll in MA plans without restrictions. CMS predicted, in the proposed rule implementing MA enrollment for dialysis patients that 26% of dialysis and transplant patients would be enrolled in MA by 2022, and current investor reports from large dialysis organizations state approximately 40% of their patients are enrolled in MA plans.^5^^,^^10^ Although approximately one-third of patients are dually eligible for Medicare and Medicaid, not all states will pay out a supplemental insurance payment so we did not account for Medicaid supplemental payment in our model nor Medicaid-only patients as they represent only 3% of the dialysis population. Instead, we relied on the 2021 unadjusted End-Stage Renal Disease Prospective Payment System base rate as a conservative Medicare fee-for-service reimbursement.^5^ Recently, Medicare fee-for-service began paying an add-on to the dialysis bundled payment for capital-related home dialysis assets known as the Capital Related Assets Transitional Add-on Payment for New and Innovative Equipment and Supplies (CRA TPNIES). CMS reimburses dialysis providers who utilize an approved home technology for CRA TPNIES 65% of the Medicare Administrative Contractor price for the equipment for a 2-year period. As of this publication, only one technology has ever been approved for CRA TPNIES based on meeting CMS substantial clinical improvement criteria for home hemodialysis treatment, effective for the 2022-2023 calendar years. Assuming use of a new home dialysis machine that that qualifies for CRA TPNIES and using the expected expenditures for TPNIES that CMS provides in the calendar year 2022 End-Stage Renal Disease Payment System final rule, analyzing Medicare 2018 dialysis facility cost report data as a baseline and adjusting for assumptions such as the reduced space required for home therapies and investment in training clinicians to support patients, we estimate $9.5 million in total health system treatment costs for this same population over 5 years, leading to a potential net operating income of $4.3 million for clinics with a home-first approach to dialysis.

**Table 2:** Economic Model a De Novo Home of a Home-First Health System Owned Dialysis Program

	Year 1	Year 2	Year 3	Year 4	Year 5	Total
**Total reimbursement**	**$2,484,370**	**$2,547,586**	**$2,813,022**	**$2,951,802**	**$3,099,476**	**$13,896,256**
Total Medicare	$1,016,156	$1,079,372	$1,104,486	$1,197,401	$1,211,772	
Total commercial	$826,105	$826,105	$944,120	$944,120	$1,062,135	
Total Medicare Advantage	$642,109	$642,109	$764,416	$810,281	$825,569	
**Total costs**	**$1,551,550**	**$1,817,734**	**$1,946,838**	**$2,023,222**	**$2,170,260**	**$9,509,604**
Total annual staffing	$327,500	$372,500	$390,750	$416,500	$412,500	
Total equipment	$288,770	$302,140	$328,770	$355,510	$368,770	
Total service	–	$164,900	$172,550	$186,150	$201,450	
Total supplies	$935,280	$978,194	$1,054,768	$1,065,062	$1,187,540	
**Total operating income**	**$932,820**	**$729,852**	**$857,842**	**$836,204**	**$921,566**	**$4,278,284**

Assuming the average health system has 50 dialysis patients needing maintenance dialysis and a primary payer mix of 20% commercial at 2.5 times the Medicare rate, 50% Original Medicare, and 30% MA at 1.14 times the Medicare rate, we estimate a health system’s dialysis treatment reimbursement could be $13.9 million for over 5 years. This includes CRA TPNIES payments in years 1-2 assuming a capital equipment MAC determined price of $42,500 depreciated over 5 years. Analyzing Medicare 2018 dialysis facility cost report data as a baseline and adjusting for assumptions such as the reduced space required for home therapies, investment in training clinicians to support patients, and investment in new capital equipment at a cost of $42,500 depreciated over 5 years, we estimate $9.5 million in total health system treatment costs for this same population over 5 years, leading to a potential net operating income of $4.3 million for clinics with a home-first approach to dialysis. CRA TPNIES, Capital Related Assets Transitional Add-on Payment for New and Innovative Equipment and Supplies; MA, Medicare Advantage; MAC, Medicare Administrative Contractor.

## Kidney Transplant

**Figure 3:**
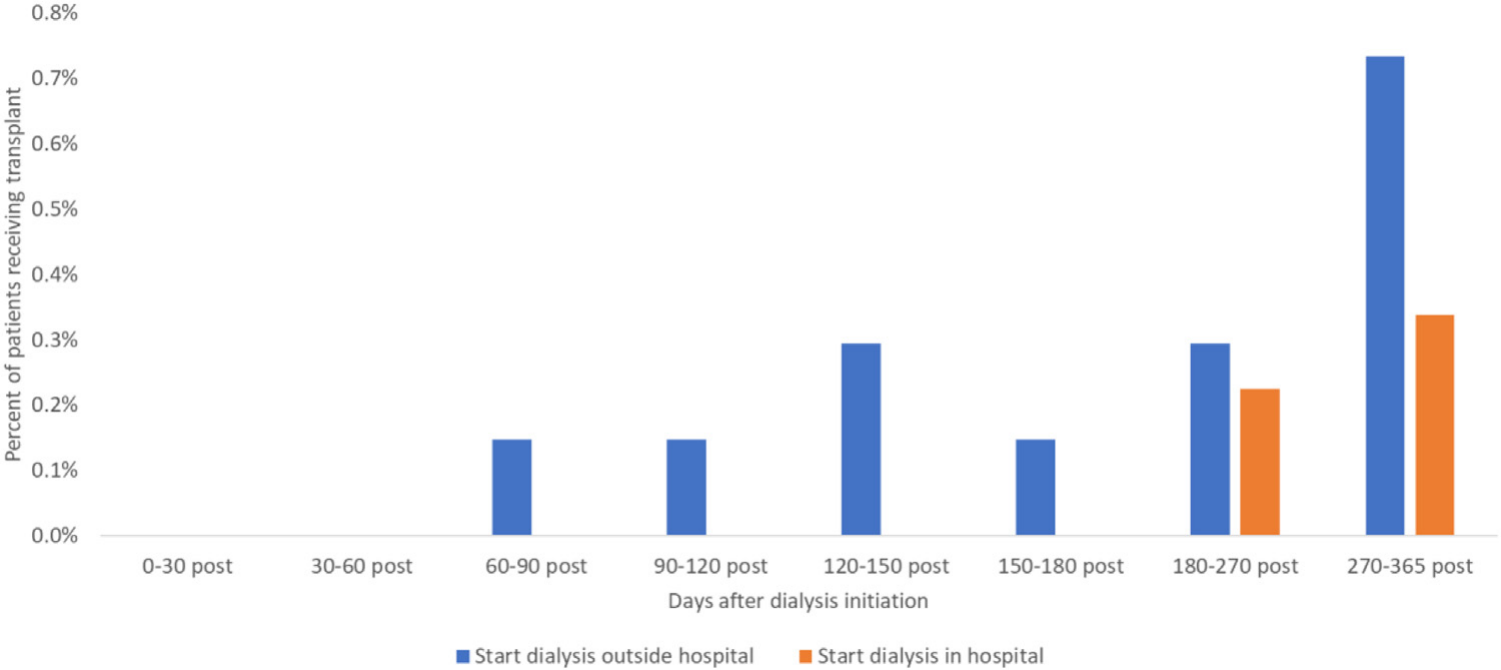
Transplant rates after dialysis initiation, Medicare patients, 2017-2018. Source: Health Management Associates analysis of 5% Medicare limited data set.

Although the total number of kidney transplants has increased, the percentage of prevalent dialysis patients receiving a kidney transplant declined from 5% to 4%, and rates among incident dialysis patients are approximately 1%.^5^ For Medicare patients who start dialysis in the hospital, the first-year transplant rate is even lower at 0.6% (Fig 3).^5^ Financial incentives for dialysis organizations currently conflict with promoting transplantation. Although the Center for Medicare & Medicaid Innovation (CMMI) is trying to change this through new value-based payment models such as the End-stage renal disease Treatment Choices (ETC) mandatory model and voluntary Kidney Care Choices (KCC) models, the ETC only measures waitlisted patients and the majority of KCC participants are run by dialysis providers who will still see the bulk of their revenues coming from their in-center dialysis business.^11^

## The Path Forward From the Status Quo in Kidney Care

Health systems with kidney transplant programs that shift towards active management of dialysis patients and accountability for kidney health may be less financially conflicted as current dialysis organizations that may attempt to move in these directions. This is because these health systems have a multitude of services across their health system to support the totality of healthcare needs for those with kidney disease. Including delaying progession of CKD and helping those with kidney failure get waitlisted for transplantation and retain active waitlist status. While capturing new revenue streams from holistic kidney care management, health systems could negotiate value-based contracts with commercial insurers, similar to Medicare demonstration models, that lower and de-risk dialysis patient costs, giving health systems greater control over the total cost of care while improving outcomes.

Recognizing the high costs and current outcomes, the Federal government has pushed kidney care into value-based care arrangements with the hopes of improving care coordination and clinical outcomes while reducing the overall spending for this population. In 2015, the CMMI introduced the first chronic specialty care model, creating End-stage renal disease Seamless Care Organizations, which centered on the voluntary participation of dialysis organizations. The 5-year End-stage renal disease Seamless Care Organizations pilot ended December 31, 2020, with interim results showing a 3% reduction in hospitalizations and a small reduction in the total cost of care without major savings to Medicare.^12^^,^^13^ Dialysis treatments delivered in the dialysis facilities increased, likely because of quality improvement efforts to improve patient adherence. There were no appreciable increases in the utilization of home dialysis or transplant. Dialysis organizations shared in Medicare savings while also benefiting from increased treatment revenue, while patient satisfaction as measured by the In-Center Hemodialysis Consumer Assessment of Healthcare Providers and Systems remained in the 50%-60% range and mortality was unchanged.^13^ These results underscore the opportunity for greater improvements beyond increasing patient adherence to in-center dialysis that might be more readily accomplished by an integrated health system that offers nephrology care.

Aforementioned, in 2019 CMMI announced its 2.0 version of kidney care demonstrations, ETC, which specifically targeted dialysis facilities and nephrologists and a second set of models collectively referred to as KCC, which provided a pathway for nephrologist-led kidney care and another for kidney care providers willing to assume risk. The mandatory ETC model randomized one-third of the country’s Medicare beneficiaries into an asymmetric model that heavily incentivizes both nephrologists and dialysis providers to increase home dialysis and transplant waitlisting.

The KCC models are piloting whether nephrologists, transplant programs, and dialysis facilities can improve management of advanced CKD to delay progression to kidney failure, improve care coordination, and increase transplantation. With nephrologists and transplant providers deemed the only required participants, CMMI appears to be signaling that kidney care models need to shift away from in-center dialysis-centric care, but the reality is that almost all of the KCCs are being run by dialysis organizations. Integrating kidney care into patients’ holistic health care needs, by focusing on population health management and leveraging new dialysis technologies, has the potential to change the kidney care trajectory. The following published case studies provide mounting evidence that population health management can improve outcomes and has been the case for health systems who have deployed intense earlier CKD care management interventions.

One health insurer’s kidney pilot program, which provided case management targeting individuals using a population stratification model within its patient-centered medical home, was associated with post-intervention reductions in hospitalizations and 30-day readmissions and achieved net savings of $481 per patient for patients with CKD class 5.^14^ Additionally, CKD management programs by a fully integrated delivery network and a hospital system in New York were associated with improved earlier CKD care and increased adoption of home dialysis and kidney transplant waitlisting for those that did progress to kidney failure.^15^^,^^16^ Indian Health Services also significantly reduced occurrence of kidney failure in patients with diabetes by 59% over 14 years through a quality improvement program focused on better diabetes and CKD management.^17^

A dialysis provider in the Northwest was an early example of how population health management strategies can be effective when performed in partnership with health system-based “transitional dialysis care programs.” In what has been referred to as a transitional program, a new dialysis patient receives care coordination that includes emotional and psychological support, in-depth disease and transplant education, nutritional counseling, care partner support, and the option to choose home dialysis.^17^

Although different types of transitional care programs in the United States are just starting to be recognized as an approach to driving more patients toward home dialysis, few have been implemented or studied.^17^, ^18^ Yet, given the strategic refresh occurring at CMMI, under the Biden Administration, with a goal to deliver more accountable care to patients, perhaps a test that could be undertaken is one that compares the outcomes of the ETC and KCC models to an intentional approach of integrating measures from these models into accountable care organizations that do not have beneficiaries aligned to ETC or KCC.

## Conclusion

Overall, the case for health systems to lead the transformation in kidney care is more compelling than ever before because of new technology and new revenue streams resulting from the expansion of dialysis patient eligibility for MA plans and reimbursement models in Medicare driving earlier kidney care management, transplantation, and home dialysis. Health systems should consider potential quality improvement and financial opportunities available to improve the holistic care of kidney patients.
